# De-Intensification of Antidiabetic Treatment Using Canagliflozin in Patients with Heart Failure and Type 2 Diabetes: Cana-Switch-HF Study

**DOI:** 10.3390/jcm10092013

**Published:** 2021-05-08

**Authors:** Luis M. Pérez-Belmonte, Michele Ricci, Jaime Sanz-Cánovas, Lidia Cobos-Palacios, María D. López-Carmona, M. Isabel Ruiz-Moreno, Mercedes Millán-Gómez, M. Rosa Bernal-López, Sergio Jansen-Chaparro, Ricardo Gómez-Huelgas

**Affiliations:** 1 Servicio de Medicina Interna, Hospital Regional Universitario de Málaga, Instituto de Investigación Biomédica de Málaga (IBIMA), Universidad de Málaga (UMA), 29010 Málaga, Spain; michele.ricci4@gmail.com (M.R.); jaimesc25@hotmail.com (J.S.-C.); cobospalacios@gmail.com (L.C.-P.); mdlcorreo@gmail.com (M.D.L.-C.); mruiz.salud@gmail.com (M.I.R.-M.); robelopajiju@yahoo.es (M.R.B.-L.); ricardogomezhuelgas@hotmail.com (R.G.-H.); 2 Servicio de Medicina Interna, Hospital Helicópteros Sanitarios, 29660 Marbella, Spain; 3 Centro de Investigación Biomédica en Red Enfermedades Cardiovasculares (CIBERCV), Instituto de Salud Carlos III, 28029 Madrid, Spain; mechimillan@hotmail.com; 4 Centro de Investigación Biomédica en Red Fisiopatología de la Obesidad y Nutrición (CIBERobn), Instituto de Salud Carlos III, 28029 Madrid, Spain

**Keywords:** de-intensification, efficacy, canagliflozin, heart failure, type 2 diabetes

## Abstract

Canagliflozin is a sodium-glucose co-transporter 2 inhibitor that reduces glycemia as well as the risk of cardiovascular events. Our main objective was to analyze antidiabetic treatment de-intensification and the glycemic efficacy of replacing antidiabetic agents (excluding metformin) with canagliflozin in patients with heart failure and type 2 diabetes with poor glycemic control. In this observational, retrospective, real-world study, we selected patients treated with metformin in combination with ≥2 non-insulin antidiabetic agents or metformin in combination with basal insulin plus ≥1 non-insulin antidiabetic agent. Non-insulin antidiabetic agents were replaced with canagliflozin. Patients were followed-up on at three, six, and 12 months after the switch and a wide range of clinical variables were recorded. A total of 121 patients were included. From baseline to 12 months, the number of antidiabetic agents (3.1 ± 1.0 vs. 2.1 ± 0.8, *p* < 0.05), basal insulin dose (20.1 ± 9.8 vs. 10.1 ± 6.5 units, *p* < 0.01), and percentage of patients who used basal insulin (47.9% vs. 31.3%, *p* < 0.01) decreased. The proportion of patients who used diuretics also declined significantly. In addition, we observed improvement in glycemic control, with an increase in the proportion of patients with glycated hemoglobin <7% from 16.8% at three months to 63.5% at 12 (*p* < 0.001). Canagliflozin use was also beneficial in terms of body weight, blood pressure, heart failure status, functional class, and cardiovascular-renal risk. There were also reductions in the number of emergency department visits and hospitalizations for heart failure. Moreover, canagliflozin was well-tolerated, with a low rate of drug-related discontinuation. Mounting evidence from randomized controlled trials and real-world studies point to the beneficial profile of sodium-glucose co-transporter type 2 inhibitors such as canagliflozin in patients with heart failure.

## 1. Introduction

Patients with heart failure (HF) frequently present with type 2 diabetes (T2D); its prevalence in this population ranges from 20% to 40% [1]. Patients with concomitant HF and T2D have worse symptoms and quality of life, greater HF hospitalization rates, and higher mortality compared to patients without T2D [2].

Poor glycemic control has been linked to increased morbidity and mortality in patients with T2D and HF who do not receive antidiabetic treatment [3]. However, once therapy is started, this association may not be linear. T2D treatment requires gradual intensification, progressively achieving glycemic targets with agents shown to be safe and effective. T2D management in patients with HF also entails controlling blood pressure, body weight, and lipids as well as preventing drug-related hypoglycemic events [4].

Renal sodium-glucose co-transporter 2 (SGLT2) inhibition causes glycosuria, which reduces hyperglycemia, as well as natriuresis, which reduces plasma volume and thus decreases systolic and diastolic blood pressure. SGLT2 inhibitors also lead to substantial weight loss, with reductions in both abdominal visceral and subcutaneous fat. Reducing fat mass could lead to declines in insulin resistance, metabolic risk, and renal risk. In addition, SGLT2 inhibitors may improve myocardial muscle efficiency and function. All these factors could explain how SGLT2 inhibitors exert cardiovascular and renal benefits beyond glycemic control [4,5]. Current guidelines recommend that SGLT2 inhibitors should be considered independently of baseline glycated hemoglobin (HbA1c) in patients with T2D and HF [6].

Canagliflozin is an SGLT2 inhibitor that has been shown to lead to better glycemic control and greater weight loss than dipeptidyl peptidase-4 (DPP4) inhibitors in patients with T2D on background metformin monotherapy [7] or metformin plus sulfonylureas [8]. Furthermore, in the CANVAS Program (CANagliflozin cardioVascular Assessment Study; and CANagliflozin cardiovascular Assessment Study-Renal)—pivotal trials on the effects of treatment with canagliflozin on cardiovascular, renal, and safety outcomes—canagliflozin significantly reduced cardiovascular and renal events as well as hospitalizations due to HF [9]. In this study, our main objective was to analyze treatment de-intensification and the glycemic efficacy of replacing antidiabetic agents (excluding metformin) with canagliflozin in patients with HF and T2D with poor glycemic control. We hypothesized that switching the antidiabetic agent to canagliflozin would simplify antidiabetic treatment and improve glycemic targets in patients with HF and T2D with poor glycemic control.

## 2. Material and Methods

### 2.1. Study Design and Patients

We carried out an observational, retrospective, real-world study on 121 outpatients with HF and T2D with poor glycemic control at the HF Unit of the Internal Medicine Department at the Hospital Regional Universitario de Málaga in Málaga, Spain, and the Hospital Helicopteros Sanitarios in Marbella, Spain.

The mean age was 64.7 ± 11.9 years (range: 44.0–84.0), and the percentage of males was 68.9%. All patients included had history of HF and T2D, HbA1c levels between 7.0% and 9.5%, and unmodified antidiabetic treatment with metformin in combination with ≥2 non-insulin antidiabetic agents or metformin in combination with basal insulin plus ≥1 non-insulin antidiabetic agent for at least three months. Patients treated with SGLT2 inhibitors and other insulin regimens were excluded. Non-insulin antidiabetic agents (excluding metformin) were replaced with canagliflozin 100 mg/day, which could be increased up to 300 mg/day during follow-up if HbA1c remained > 7% or the healthcare providers considered it appropriate according to their clinical judgment. All patients received general recommendations during follow-up for following a healthy diet and doing physical activity according to their functional class. Antihypertensive treatment, diuretics, and lipid-lowering agents were modified if required as per the healthcare providers’ judgment.

Patients were followed-up on at 3, 6, and 12 months after starting canagliflozin. A wide range of sociodemographic, anthropometric (body weight, body mass index (BMI), and waist circumference), clinical (T2D duration and therapy, HF duration, principal cause, left ventricular ejection fraction, fractional shortening and medication, and previous medical history including history of smoking, history of alcohol abuse, hypertension, dyslipidemia, chronic kidney disease stage ≥ 3, cerebrovascular disease, chronic obstructive pulmonary disease and atrial fibrillation), therapeutic (any reduction in doses or number of HF medications), and laboratory variables (basal fasting blood glucose; serum creatinine; estimated glomerular filtration rate measured using the Chronic Kidney Disease Epidemiology Collaboration (CKD-EPI) formula; uric acid; hematocrit; LDL, HDL, and total cholesterol; triglycerides; N-terminal pro-brain natriuretic peptide (NT-proBNP); and urinary albumin/creatinine ratio) was recorded at each evaluation. Other variables gathered after switching were HF health status, estimated using the total symptom score on the Spanish version of the Kansas City Cardiomyopathy Questionnaire (KCCQ) [10]; vascular risk, estimated using the Framingham equation adapted for the Spanish population (Registre Gironí del COR (Girona Heart Registry)), the REGICOR Study [11]); fatty liver disease, estimated using the Fatty Liver Index (FLI) [12]; adverse drug reactions; hypoglycemic episodes, as defined by the American Diabetes Association criteria [13]; need for canagliflozin discontinuation due to adverse events; 3-point major adverse cardiovascular events (3P-MACE) (composite of nonfatal stroke, nonfatal myocardial infarction, and cardiovascular death); emergency department visit due to HF; hospitalizations (all-cause and for HF); and mortality (for cardiovascular and non-cardiovascular causes).

Investigators reviewed each patient’s electronic medical record in order to collect patient data. Written informed consent for consulting patient medical records was obtained from all participants. The study was approved by the Institutional Research Ethics Committee of Málaga (Ethics Committee code: CANA-HF-22-03-18) and conducted in accordance with the Declaration of Helsinki.

### 2.2. Study Outcomes

The primary endpoint was to analyze de-intensification of antidiabetic treatment (reduction in number of antidiabetic agents and/or doses of basal insulin) and glycemic control (reduction in levels of HbA1c and proportion of patients who achieved good glycemic control, defined as HbA1c < 7%) before initiating canagliflozin and at 3, 6, and 12 months. Secondary endpoints were to analyze changes in body weight; BMI; waist circumference; blood pressure levels; heart rate; HF health status estimated using both the total symptom score on the Spanish version of the KCCQ and the New York Heart Association (NYHA) functional class; HF medications (antihypertensive agents, beta-blockers, and diuretics); vascular risk; fatty liver disease; laboratory variables; adverse drug reactions; major cardiovascular events (3P-MACE); urgent HF visit (from one year before switching); hospitalizations (from one year before switching); and mortality after switching.

### 2.3. Statistical Analysis

Statistical analyses were performed using SPSS Statistics for Windows, version 15.0. Quantitative variables were expressed as means ± standard deviation and qualitative variables as absolute values and percentages. Student’s *t*-test and the repeated measures analysis of variance were used to compare quantitative variables whereas Pearson’s chi-square and McNemar’s test were used for qualitative variables. Values were considered to be statistically significant when *p* < 0.05.

## 3. Results

A total of 121 patients were included in the study. Treatment with metformin in combination with two non-insulin antidiabetic agents was the most frequently used regimen before the switch (43.8%), followed by metformin in combination with basal insulin plus one non-insulin antidiabetic agent (37.2%), metformin in combination with basal insulin plus two non-insulin antidiabetic agents (10.7%), and metformin in combination with three non-insulin antidiabetic agents (8.3%). Baseline sociodemographic, clinical, and treatment characteristics are shown in Table 1. After switching, all patients started with canagliflozin 100 mg/day. Canagliflozin was increased to 300 mg/day in 58 patients (48.7%) at three months and in 79 patients (68.1%) at six months.

From baseline to 12 months, there was a reduction in the number of antidiabetic agents (3.1 ± 1.0 to 2.1 ± 0.8 agents (*p* < 0.05)) and a progressive decline in the basal insulin dose (20.1 ± 9.8 to 10.1 ± 6.5 units (*p* < 0.01)); these changes were already significant at three months. The percentage of patients who used basal insulin decreased from 47.9% at baseline and three months to 36.2% at six months (*p* < 0.01) and 31.3% at 12 months (*p* < 0.01). The percentage of patients who used diuretics was also significantly lower at 6 and 12 months. There were no other significant changes in other medications.

In regard to glycemic control, there were significant reductions in fasting blood glucose (BG) and HbA1c from three months’ follow-up. The proportion of patients with HbA1c < 7% significantly increased, from 16.8% of patients at three months to 63.5% at 12 months (*p* < 0.001).

During follow-up, patients treated with canagliflozin experienced weight loss, with significant reductions in BMI, proportion of patients with BMI ≥ 30, and waist circumference. Systolic and diastolic blood pressure levels also declined whereas there were no significant changes in heart rate.

KCCQ total symptom score progressively increased from baseline to 12 months’ follow-up (62.2 ± 24.8 vs. 75.9 ± 28.0, *p* < 0.01). There was also an improvement in NYHA functional class, with fewer patients in class III after switching and reaching its lowest point at 12 months (15.7% vs. 38.0% at baseline, *p* < 0.01). Vascular risk also decreased during follow-up, from 18.9 ± 12.4 to 8.5 ± 5.1 (*p* < 0.001), as did the FLI, from 79.9 ± 22.1 to 63.0 ± 13.2 (*p* < 0.01).

Significant differences from baseline to 12 months were observed for different laboratory variables: uric acid (−0.9 mg/dL, *p* < 0.05), LDL cholesterol (−17.8 mg/dL, *p* < 0.01), HDL cholesterol (+6.4 mg/dL, *p* < 0.05), total cholesterol (−29.7 mg/dL, *p* < 0.01), triglycerides (−28.9 mg/dL, *p* < 0.01), NT-proBNP (−565.5 pg/mL, *p* < 0.01), and urinary albumin/creatinine (−45.5 mg/g, *p* < 0.01).

In regard to safety variables, 15 patients (13.0%) had an adverse drug reaction of interest as of the end of follow-up (7 urinary tract infections and 8 genital mycotic infections). This led to canagliflozin treatment being suspended in 6 patients (5.0%). The remaining adverse drug reactions were successfully resolved with antimicrobial treatment. In addition, there were four 3P-MACEs linked to four cardiovascular deaths. Emergency department visits and hospitalizations due to HF were significantly lower 12 months after switching compared to baseline (45 vs. 61, *p* < 0.05 and 31 vs. 48, *p* < 0.05, respectively).

All data on glycemic control, treatment simplification, anthropometric characteristics, HF health status, vascular risk, fatty liver disease, laboratory variables, adverse drug reactions, and major complications are summarized in Table 2. Similar results were observed in the study outcomes when patients were grouped according to mean age (<65 vs. ≥65 years old) and sex (male vs. female).

## 4. Discussion

Our real-world study found that in patients with HF and T2D with poor glycemic control, switching from non-metformin oral antidiabetic agents to canagliflozin simplified treatment led to reductions in the number of antidiabetic agents, basal insulin doses, and percentage of patients who use diuretics and led to improved glycemic control, with reductions in BG and HbA1c levels. In addition, the use of canagliflozin had a favorable cardio-renal-metabolic profile in regard to body weight, blood pressure, HF health status, functional class, and cardiovascular-renal risk. There were also reductions in the number of emergency department visits and hospitalizations due to HF.

HF is a complex, chronic condition which is frequently associated with concomitant chronic conditions, including T2D [14]. As a result of these associations, patients with HF are normally subjected to polypharmacy, making the therapeutic approach very complex [15]. For patients with HF, the use of SGLT2 inhibitors is recommended as part of the glucose-lowering regimen due to their cardiovascular and renal benefits, which go beyond glycemic control [4,5]. On the other hand, patients with a complex health status are more likely to have adverse drug reactions [16]. Therefore, implementing a treatment plan with the goal of de-intensifying antidiabetic treatment by switching antidiabetic agents to SGLT2 inhibitors could be beneficial in patients with HF and T2D. In our study, replacing antidiabetic agents with canagliflozin reduced the number of antidiabetic agents used (from 3.1 before initiating canagliflozin to 2.1 at 12 months’ follow-up), the basal insulin dose (from 20.1 units/day to 10.1 units/day), and the proportion of patients treated with basal insulin (from 47.9% to 31.3%). Furthermore, we observed an improvement in the glycemic profile, with a reduction in HbA1c of 1.2% and an increase in the percentage of patients who achieved an HbA1c <7% to 63.5% at one year after the switch.

In a previous 26-week study (SITA-CANA Switch Study) [17], switching from sitagliptin (and, where appropriate, gliclazide as well) to canagliflozin 100 or 300 mg/day reduced HbA1c, weight, and systolic and diastolic blood pressure, which is in line with our results. However, the proportion of patients who achieved HbA1c <7% was higher in our study (63.5%) compared to 42% of patients in the SITA-CANA Switch Study. The differences in treatment de-intensification and glycemic control between this study and the SITA-CANA Switch Study could be explained by a longer follow-up period after the switch (12 months vs. 6 months); the general recommendations on diet and physical activity provided in this study; and the close management of these patients, who belonged to the HF Unit of the Internal Medicine Department.

These factors may also explain the differences in results in our work compared to the CANVAS Program in regard to reductions in HbA1c, weight, and systolic and diastolic blood pressure [8]. In our study, the reduction observed in HbA1c was greater than in the CANVAS Program (1.2% vs. 0.6% at 12 months of follow-up). Regarding weight, patients lost an average of 5 kg at the end of the follow-up period, which is greater than the weight loss described in the CANVAS trials, which was around 3 kg. It is possible that the high proportion of patients treated with sulfonylureas, the lower number of patients on basal insulin, or the reduction in basal insulin dose could justify the greater amount of weight lost in our study. In our study, other antidiabetic agents that can trigger weight loss, such as glucagon-like peptide-1 (GLP-1) receptor agonists [18], could have been added to the antidiabetic treatment if the healthcare provider deemed it appropriate. Nevertheless, GLP-1 receptor agonists were initiated in only one patient and thus did not significantly influence this result.

SGLT2 inhibitors have been found to interfere in the lipid profile, increasing LDL and HDL cholesterol levels and reducing triglyceride levels [19]. It has been suggested that the change in the lipid profile is due to reduced LDL particle clearance from circulation and increased lipolysis of triglyceride-rich proteins [20]. In our study, there were reductions in total and LDL cholesterol levels as well as triglycerides and an increase in HDL cholesterol levels. These changes were greater than those described in previous studies [19] and are likely due to the close monitoring of these patients, which included recommendations on diet and physical activity. The improvements in blood pressure and lipid profiles positively impacted these patients’ vascular risk [10] and improved renal outcomes, as evidenced by a decrease in the urinary albumin/creatinine ratio. In addition, the fatty liver index also improved. It is known that nonalcoholic fatty liver disease is common in patients with T2D and that SGLT2 inhibitors are capable of reducing the liver’s fat content and improving the biological markers of hepatic steatosis [21].

Several randomized clinical trials have analyzed the effect of SGLT2 inhibitors on glycemic control and cardiovascular and renal outcomes in patients with T2D [9,22,23,24,25,26]. All of them found a consistent benefit in terms of hospitalization for acute decompensated HF in patients with reduced and preserved ventricular ejection fraction [24,27]. These results have also been described in real-world studies [28,29,30,31,32]. These benefits in HF seem to be independent of the hypoglycemic effect of SGLT2 inhibitors and have been linked to an increase in urinary sodium excretion and a reduction in body fluids, weight, and blood pressure [33,34]. Their effect on red blood cell concentration and uric acid has also been described as having a potentially beneficial outcome on HF hospitalizations [35]. In our study, there were significant reductions in number of emergency department visits and hospitalizations due to HF at 12 months after the switch as well as an increase in hematocrit levels and a reduction in uric acid levels. Despite these reductions, the percentages of emergency department visits and hospitalizations due to HF at 12 months after initiation of canagliflozin were still high (39.1% and 27.0%, respectively). The use of canagliflozin also resulted in a reduction of NT-proBNP levels and an improvement in HF symptoms, as measured by the KCCQ, and HF functional class, as measured by the NYHA classification.

Due to their natriuretic and diuretic effects, SGLT2 inhibitors reduce plasma volume and decrease cardiac preload [36]. In addition, they have the ability to inhibit sodium-hydrogen exchangers in the heart and kidneys, which could amplify the natriuretic effects of other drugs commonly administered to patients with HF (loop diuretics and mineralocorticoid receptor antagonists) [37]. This could allow for a reduction in the percentage of patients with HF who receive diuretic treatment. In our study, around 10% of patients had discontinued diuretics by 12 months’ follow-up.

In regard to safety variables, the number of adverse drug reactions of interest with canagliflozin in our study was small. All were related to genitourinary infections and were mostly minor. Only 5% of patients discontinued canagliflozin treatment because of them. No significant increase in major complications was observed in our study.

Although our findings are important, we acknowledge several potential limitations. First, the observational nature of our data, the lack of a control group, and the limited number of patients may have led to bias and limit the extrapolation of our results. Second, due to the low number of events or complications, their relationship to canagliflozin use could not be conclusively determined. Third, given that antihypertensive treatment, diuretics, and lipid-lowering agents could be modified if deemed appropriate according to healthcare providers’ criteria and patients received general recommendations on following a healthy diet and physical activity according to their functional class during follow-up, we cannot strictly attribute all results to the change in antidiabetic drugs to canagliflozin. Finally, in our study, only canagliflozin was evaluated. Thus, our findings cannot be extrapolated to other SGLT2 inhibitors.

## 5. Conclusions

In conclusion, our real-world study found that switching non-metformin oral antidiabetic agents to canagliflozin simplified antidiabetic treatment, reduced the percentage of patients who use diuretics, and improved glycemic control in patients with HF and T2D with poor glycemic control. In addition, the use of canagliflozin showed a favorable cardiovascular, renal, and metabolic profile, with reductions in emergency department visits and hospitalizations due to HF and improvements in HF health status and functional class. Canagliflozin was also well-tolerated and there was a low rate of drug-related discontinuation. Mounting evidence from randomized controlled trials and real-world studies such as this one point to the beneficial profile of SGLT2 inhibitors in patients with HF.

## Figures and Tables

**Table 1:** Baseline sociodemographic and clinical-therapeutic characteristics.

Variables	*n* = 121
Sociodemographic characteristics	
Age (years)	64.7 ± 11.9
Male	83 (68.9%)
Diabetes characteristics	
Diabetes duration (years)	13.5 ± 4.8
Diabetes therapy	
Metformin	108 (89.3%)
Sulfonylurea	45 (37.2%)
Meglitinide	15 (12.4%)
Thiazolidinediones	0
DPP4 inhibitor	110 (90.9%)
GLP-1 receptor agonist	11 (9.1%)
Basal insulin	58 (47.9%)
Statins	109 (90.1%)
Heart failure characteristics	
Heart failure duration (years)	4.5 ± 2.1
Principal cause of heart failure	
Ischemic	70 (57.9%)
Nonischemic	42 (34.7%)
Unknown	9 (7.4%)
Left ventricular ejection fraction (%)	44.1 ± 10.1
Left ventricular ejection fraction < 40%	58 (47.9%)
Fractional shortening (%)	21.9 ± 7.8
Heart failure medication	
Diuretic	110 (90.9%)
ACE inhibitor	51 (42.1%)
ARB	30 (24.8%)
Sacubitril-valsartan	40 (33.1%)
Beta-blocker	101 (83.5%)
Mineralocorticoid receptor antagonist	68 (56.2%)
Digitalis	12 (9.9%)
Previous medical history	
History of smoking	63 (52.1%)
History of alcohol abuse	31 (25.6%)
Hypertension	108 (89.3%)
Dyslipidemia	102 (84.3%)
Chronic kidney disease stage ≥ 3	31 (25.6%)
Cerebrovascular disease	13 (10.7%)
Chronic obstructive pulmonary disease	48 (39.7%)
Atrial fibrillation	39 (32.2%)

Continuous data are shown as means (standard deviations) and qualitative data as absolute value and percentage. ACE: angiotensin-converting enzyme; ARB: angiotensin receptor blocker; DPP4: dipeptidyl peptidase-4; GLP-1: glucagon-like peptide-1.

**Table 2:** Treatment de-intensification, glycemic control, anthropometric characteristics, heart failure health status, vascular risk, fatty liver disease, laboratory variables, adverse drug reactions, and major complications.

Variables	Baseline (*n* = 121)	3 Months’ Follow-Up (*n* = 119)	6 Months’ Follow-Up (*n* = 116)	12 Months’ Follow-Up (*n* = 115)
Treatment de-intensification				
Number of antidiabetic agents	3.1 ± 1.0	2.1 ± 0.9 *	2.1 ± 0.9 *	2.1 ± 0.8 *
GLP-1 receptor agonist	11 (9.1%)	12 (10.1%)	12 (10.3%)	12 (10.4%)
Basal insulin dose (Units/day)	20.1 ± 9.8	16.6 ± 8.8 *	12.8 ± 7.1 ^†^	10.1 ± 6.5 ^†^
Basal insulin	58 (47.9%)	57 (47.9%)	42 (36.2%) ^†^	36 (31.3%) ^†^
Diuretic	110 (90.9%)	105 (88.2%)	95 (81.9%) *	93 (80.9%) *
Glycemic control				
Fasting blood glucose (mg/dL)	157.8 ± 41.3	141.8 ± 62.8 ^†^	122.8 ± 47.4 ^‡^	118.7 ± 40.1 ^‡^
HbA1c (%)	8.1 ± 0.8	7.6 ± 1.2 *	7.1 ± 1.3 ^†^	6.9 ± 1.2 ^†^
Patients with HbA1c < 7%	-	20 (16.8%) *	58 (50%) ^‡^	73 (63.5%) ^‡^
Anthropometric characteristics				
Body weight (kg)	88.7 ± 14.3	86.8 ± 13.0	84.7 ± 12.4 *	83.4 ± 11.2 ^†^
Body Mass Index (kg/m^2^)	32.4 ± 5.6	31.5 ± 4.5	30.2 ± 4.0 *	29.2 ± 3.7 ^†^
Body Mass Index ≥ 30	51 (42.1%)	46 (38.7%)	40 (34.5%) *	34 (29.6%) ^†^
Waist circumference (cm)	112.1 ± 15.4	109.0 ± 12.1	105.2 ± 11.4 ^†^	103.1 ± 10.1 ^†^
SBP (mmHg)	141.1 ± 17.6	138.5 ± 12.9	135.4 ± 10.9	133.5 ± 10.5
DBP (mmHg)	73.9 ± 9.2	71.2 ± 8.2	69.4 ± 7.9	68.5 ± 7.5
Heart rate (bpm)	69.6 ± 7.4	70.0 ± 7.9	65.3 ± 6.8	68.6 ± 7.2
HF health status				
KCCQ total symptom score	62.2 ± 24.8	69.1 ± 25.3	72.2 ± 26.8 *	75.9 ± 28.0 ^†^
NYHA functional class				
I	0	5 (4.2%)	6 (5.2%)	6 (5.2%)
II	75 (62.0%)	84 (70.6%) *	86 (74.1%) ^†^	91 (79.1%) ^†^
III	46 (38.0%)	30 (25.2%) *	24 (20.7%) ^†^	18 (15.7%) ^†^
Vascular risk	18.9 ± 12.4	12.9 ± 6.9 *	10.3 ± 5.8 *	8.5 ± 5.1 ^†^
Fatty liver index	79.9 ± 22.1	70.2 ± 16.7 *	68.8 ± 15.1 *	63.0 ± 13.2 ^†^
Laboratory variables				
Creatinine (mg/dL)	0.93 ± 0.41	0.82 ± 0.44	0.85 ± 0.43	0.83 ± 0.43
EGFR (ml/min/1.73 m^2^)	75.8 ± 16.2	71.3 ± 19.1	73.2 ± 18.1	76.9 ±18.7
Uric acid (mg/dL)	6.4 ± 1.6	6.0 ± 2.0	6.0 ± 1.3	5.5 ± 1.2 *
Hematocrit (%)	30.0 ± 5.8	31.1 ± 5.9	32.2 ± 6.1	33.8 ± 7.0 *
LDL cholesterol (mg/dL)	84.5 ± 28.5	68.5 ± 21.4 ^†^	68.2 ± 21.0 ^†^	66.7 ± 20.1 ^†^
HDL cholesterol (mg/dL)	37.0 ± 11.5	38.3 ± 10.4	40.4 ± 10.2	43.4 ± 11.2 *
Total cholesterol (mg/dL)	159.0 ± 33.2	145.0 ± 29.3 *	144.1 ± 30.0 *	129.3 ± 26.7 ^†^
Triglycerides (mg/dL)	187.7 ± 49.9	183.0 ± 42.5	175.8 ± 38.3 *	158.8 ± 31.5 ^†^
NT-proBNP (pg/mL)	1175.5 ± 423.1	636.0 ± 452.3 ^†^	645.2 ± 432.1 ^†^	610.0 ± 398.2 ^†^
Urinary albumin/creatinine ratio (mg/g)	59.8 ± 19.3	32.5 ± 10.0 ^†^	24.7 ± 9.7 ^†^	14.3 ± 6.2 ^†^
Safety variables ^a^				
Adverse drug reaction of interest	-	8 (6.6%)	12 (8.3%)	15 (12.4%)
Urinary tract infections	-	3	5	7
Genital mycotic infections	-	5	7	8
Discontinuation of canagliflozin	-	2 (1.7%)	5 (4.1%)	6 (5.0%)
Major complication ^a^				
3P-MACE	-	0	3 (2.5%)	4 (3.3%)
Emergency department visit due to HF	61 (50.4%)	12 (10.1%)	24 (20.7%)	45 (39.1%) *
Hospitalization				
Due to HF	48 (39.7%)	11 (9.2%)	17 (14.7%)	31 (27.0%) *
All-cause	10 (8.3%)	0	0	2 (1.7%) *
Mortality	-			
Cardiovascular cause	-	0	3 (2.5%)	4 (3.3%)
Non-cardiovascular cause	-	0	0	1 (0.8%)
HF hospitalization and cardiovascular mortality	-	11 (9.2%)	20 (17.2%)	35 (30.4%)

Continuous data are shown as means (standard deviations) and qualitative data as absolute value and percentages. Statistical significance was measured for the comparison of baseline and follow-up data. DBP: diastolic blood pressure; EGFR: estimated glomerular filtration rate; GLP-1: glucagon-like peptide-1; HbA1c: glycated hemoglobin; HF: heart failure; KCCQ: Kansas City Cardiomyopathy Questionnaire; 3P-MACE: 3-Point major adverse cardiovascular event; NT-proBNP: N-terminal pro-brain natriuretic peptide; NYHA: New York Heart Association; SBP: systolic blood pressure. ^a^ Cumulative data during the 12 months of follow-up are shown. * *p* < 0.05; ^†^ *p* < 0.01; ^‡^ *p* < 0.001.

## Data Availability

Not applicable.
